# Effect of Load Cycling on the Fracture Strength/Mode of Teeth Restored with FRC Posts or a FRC Liner and a Resin Composite

**DOI:** 10.1155/2018/9054301

**Published:** 2018-08-14

**Authors:** Maria D. Gaintantzopoulou, Eleftherios T. Farmakis, George C. Eliades

**Affiliations:** Department of Biomaterials, School of Dentistry, National and Kapodistrian University of Athens, 11521 Athens, Greece

## Abstract

The aim of the study was to comparatively evaluate the fracture strength and mode of root canal treated teeth restored with resin composites with and without posts. The lingual cusps of root canal treated first upper premolars (n = 10/group) were removed down to cervical enamel and restored with the following: group A: glass-fiber post (Glassix) followed by a particulate-filled composite resin (PFC, G-aenial posterior, 3 × 2 mm layers); group B: glass-fiber reinforced composite bulk fill liner (EverX posterior, 4 mm layer) with the PFC (2 mm layer). Specimens were immersed in H_2_O (1 w/37°C), then subjected to load cycling (50 N/0.2 Hz/200k cycles), and fractured under compressive loading. Failure mode was characterized by stereomicroscopy. Statistical analysis was performed by Mann-Whitney (load) and Chi-square (mode) at a = 0.05. No statistically significant differences (p = 0.273) were found in fracture load between median values of groups A (860 N) and B (1059 N). In group A, 60% of the specimens demonstrated catastrophic root fractures and 40% mixed crown fractures (tooth cusp and restoration), whereas in group B, no root fractures were found, and the failure modes were equally distributed between mixed fractures as above and fracture of the buccal cusp. These differences were statistically significant (p = 0.004). The combination of the glass-FRC bulk fill liner with the PFC diminished the catastrophic root fractures induced by FRC posts, at a similar or higher fracture load.

## 1. Introduction

The strength and longevity of the endodontically treated teeth (ETT) are still a controversial issue of high concern for clinicians and researchers. Long-term survival rate of ETT not only depends on the success of the endodontic treatment, since the remaining tooth structure and the definitive restoration are determinant factors, as well [1]. Restoring ETT with appropriate materials and techniques, capable of resisting fracture, is of paramount importance. With the development of new adhesive materials and techniques, these structurally compromised teeth could be reinforced.

Endodontically treated teeth often require post and core restorations for retention purposes, because of extensive loss of tooth structure due to caries or fracture. However, it has been demonstrated that cast and prefabricated metallic posts do not strengthen the tooth and do not improve ETT longevity [2–4]. As an alternative, fiber-reinforced composite (FRC) posts have been developed with a modulus of elasticity matching that of human dentin, resulting in a more even stress distribution along the root and therefore in less incidence of catastrophic failures [5–8]. Moreover, glass-FRC posts and composite resin core built-up materials demonstrate improved esthetics, when semitransparent materials are considered for the main restoration.

Although enhanced restoration retention and favorable distribution of the occlusal forces along the remaining tooth structure are the major functions of FRC posts [9–11], several disadvantages and limitations have been associated with their clinical use. Interestingly, based on a meta-analysis of clinical studies, the overall rate of catastrophic failures between metal and FRC posts was similar, with the prefabricated metal and carbon-FRC posts demonstrating a two-time higher failure incidence from cast metal and glass-FRC posts [12].

The importance of the remaining amount of coronal tooth structure and intracanal dentin wall thickness on the fracture resistance of endodontically treated teeth have been already emphasized in previous studies [13–15]. Considering that post space preparation has been shown to weaken the remaining tooth structure [16, 17], it would be tempting if post placement could be omitted, introducing new minimally invasive therapeutic options [18, 19]. Since direct composite restorations may not function optimum in ETT with extended tooth structure loss, indirect onlay, overlay, or endocrown bonded ceramic restorations have been suggested as more conservative approaches to post and core and full coverage restorations for badly broken ETT, without the need for aggressive macroretentive preparation [20–23]. However, catastrophic failures below the cementoenamel junction (CEJ) have been reported even with conservative onlays or endocrowns. As an alternative to ceramic indirect restorations, polymer composites have been proposed due to their superior stress-absorbing properties [22, 24, 25].

Recently a glass-FRC resin composite has been introduced to be used as a bulk liner for direct particulate-filled resin composite (PFC) restorations. This material is reinforced with short glass fibers (diameter 12-17 *μ*m, length 0.3-1.9 mm, and critical fiber length 0.85-1.09 mm) randomly distributed in a conventional light-cured dimethacrylate resin matrix, along with particulate fillers [26]. The fiber reinforcing mechanism is based on the principle that a relatively soft ductile polymer matrix may transfer an applied load to the fibers via shear forces at the interface [27]. Therefore, a short glass-FRC bulk fill liner can be applied in a single-layer and serve as a reliever to polymerization stresses [28], improving the mechanical performance of the tooth-restoration structural complex [29–31].

The aim of the study was to comparatively evaluate the strength and fracture mode of root canal treated teeth restored with a resin composite, employing a root canal glass-FRC post or a glass-FRC bulk fill liner, after load cycling. The null hypothesis was that there are no differences in the fracture strength and failure mode among the two restorative modalities tested.

## 2. Materials and Methods

**Figure 1:**
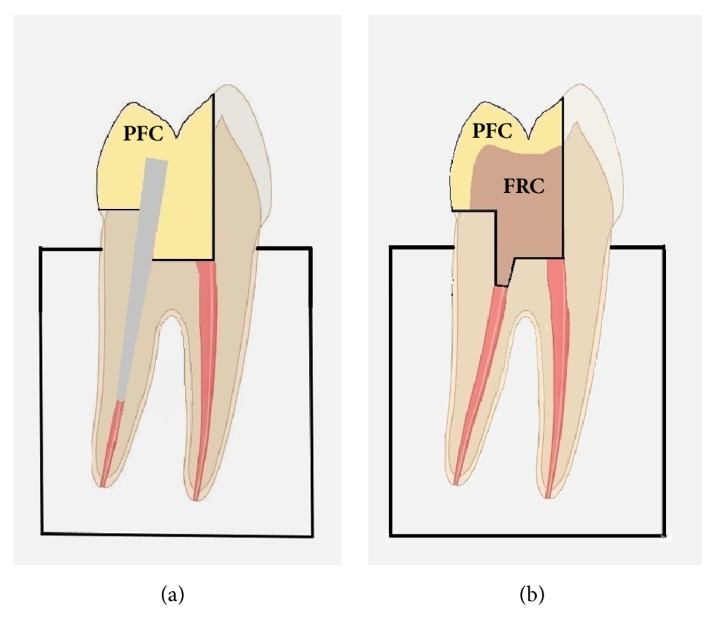
Schematic illustration of the test specimens. PFC refers to the particulate filler composite and FRC to the short glass-fiber reinforced bulk fill liner. (a) Group A: glass-fiber-reinforced posts and PFC; (b) Group B: FRC liner and PFC.

First upper premolars (#14 and 24, all intact with two fully developed roots), extracted for orthodontic reasons and kept in distilled water with 0.5% sodium azide at 8°C, were used in the study. The use of this material was approved by the Ethics Committee of the institution (#265b/30.3.2015). The teeth selected were of similar crown and root sizes and with no cracks or other defects as examined under a stereomicroscope (M80, Leica Microsystems, Wetzlar, Germany) at 4X magnification. The teeth were randomly distributed into two groups (A-B, n = 12 each) and subjected to root canal treatments. Access cavity was prepared by #330 and EndoZ burs (Dentsply-Maillefer, Ballaigues, Switzerland), and working length was determined for each canal as 1 mm short of the length of No 10 K-file (Dentsply-Maillefer), just protruding the apical foramen. For canal preparation, the Protaper Universal System (Dentsply-Maillefer) was applied up to F3 instrument. In between each file, 5 ml of 2.5% NaOCl irrigating solution was used; canal was dried with high vacuum aspiration and a small quantity of 18.6% EDTA lubricating gel (Ultradent, South Jordan, Utah, USA) was placed in the canal, proceeding the next file. Following completion of the root canal preparation, smear layer was removed by 10 ml REDTA 17% solution (Roth Int, Chicago, Ill, USA), and rinsing was completed by 10 ml sterile saline. All root canals were dried with paper points and subsequently obdurated by cold lateral condensation of gutta-percha points (Hygenic, Coltene/Whaledent Langenau, Germany) and an epoxy based sealer (AH Plus, Dentsply DeTrey GmbH, Konstanz, Germany). Excess gutta-percha was removed at the orifice of the canal with a hot instrument and the access cavity was provisionally filled with a cotton pellet and a temporary filling material (Caviton, GC International, Tokyo, Japan). All specimens were stored in 100% humidity and 37°C for a week to allow for full sealer setting. Then, the palatal cusp of each premolar was removed up to 0.5 mm length from the cervical enamel margin with a cylindrical diamond bur (Komet Dental, Lemgo, Germany) attached to an air-rotor handpiece and the teeth were restored with the materials listed in Table 1 as follows:

For group A (Figure 1(a)), the temporary filling material and cotton pellet were removed and a size 1 Peeso reamer (Dentsply, Maillefer, Tulsa, OK) was used to remove the filling material from the lingual root canal up to 8 mm depth from the cut cervical enamel. A glass-FRC post was silanated with the silane primer, left intact for 60 s, air-dried for 10 s, and then cemented into the prepared root canal with the self-adhesive luting agent, which was light-cured for 20 s. The post length used for retention of the restorative material was approximately 3 mm. The enamel margins were etched with the phosphoric acid gel for 10 s, rinsed with water for 5 s, and gently air-dried for 5 s, then the adhesive was applied over the prepared tooth and post surfaces exposed, left undisturbed for 10 s, air-dried for 5 s, and light-cured for 10 s. For the final restoration the PFC posterior restorative was applied (3 × 2 mm increments) and each increment was light-cured for 40 s. Finally, the restoration was contoured, finished, and polished with composite finishing carbide burs (Komet Dental) and alumina polishing discs (Soflex, 3 M ESPE, St. Paul, MN, USA).

For group B (Figure 1(b)), removal of the filling material from the lingual root canal was limited to a 2 mm depth from the cut cervical enamel. Acid-etching of enamel margins and adhesive application were performed as before. Then, the glass-FRC bulk fill liner was applied in a single 4 mm increment, including intracanal extension and light-cured for 40 s. A final layer of the PFC restorative (1 × 2 mm increment) was placed, light-cured, contoured, and finished as above. In all cases, light-curing was performed with a LED unit (G2 Bluephase, Ivoclar Vivadent) with a curing distance of 0.5 mm, operating at high mode (1200 mW/cm^2^ light intensity). Specimens were inspected under the stereomicroscope for presence of marginal defects. Two specimens were discarded from each group, creating thus two groups of 10 specimens each.

To succeed proper alignment of the loading device with each occlusal tooth surface, the root apices of each specimen were cut and fixed at the bottom of empty cylindrical transparent plexiglass molds (Ø:15 mm, h: 15 mm), which were placed in-line with the stainless steel sphere (Ø: 5 mm) of the loading device. The external plexiglass surface was marked with 3 notches relative to the base of the loading device to guarantee proper repositioning of the molds. The molds were then filled with fast setting acrylic resin (Kallocryl CP GM, Dr Speier GmbH, Münster, Germany) up to a 2 mm distance apically to the dentine-enamel junction. Care was taken to avoid porosity in the embedding material, by inspection of the pouring resin through the transparent plexiglass molds. In this way individual aligned bases were produced for each specimen. All specimens were immersed in distilled water for 1 week at 37°C and then prepared for the load cycling testing.

**Figure 2:**
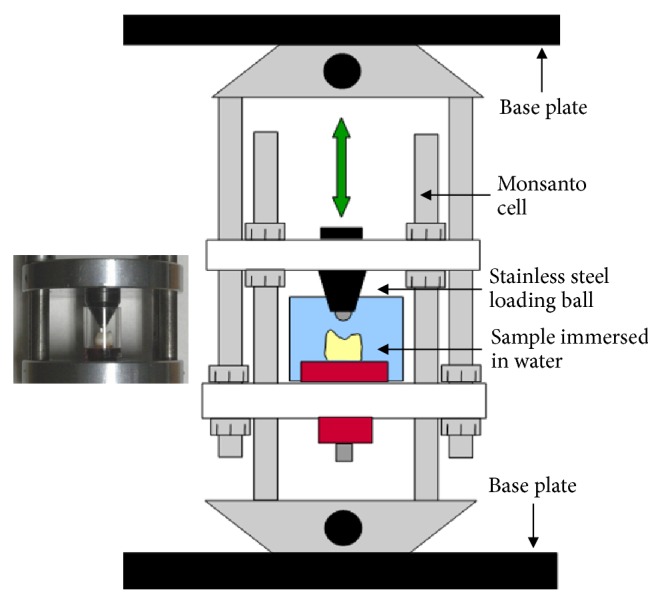
The setup used for specimen load cycling.

Each specimen was placed in the loading cell (Monsanto compression cell), of a custom made load cycling unit, aligned and subjected to load cycling for 200,000 cycles at 0.2 Hz, under vertical movement of the loading head (Figure 2). A 50 N load was applied at the inclined surfaces of the premolar occlusal cusps, in contact with tooth walls and restoration surface. During load cycling the tooth crown and the loading sphere were kept in a water-cell at ambient temperature (25°C). Following load cycling, the specimens were stored again in distilled water for 1 week at 37°C and then loaded up to fracture in a universal testing machine (Model 6022, Instron, Canton, MA, USA) equipped with a similar cell at a crosshead speed of 0.5 mm/min. The fracture load was recorded in Newtons (N).

The failure mode of all the specimens was characterized by stereomicroscopy; at 7X the mode of failure was characterized as type I (failure of the buccal tooth crown wall), type II (failure of the restoration), type III (combination of type II and III failures), and type IV (root fracture).

Statistical analysis of the failure load (in N) was performed by the Mann-Whitney Rank Sum Test. For the failure mode, percentage frequencies were compared by the Chi-square test. All tests were performed with SigmaStat v 3.1 software (Jandel, S. Raphael, Ca, USA) at a 95% confidence level (a = 0.05).

## 3. Results

**Figure 3:**
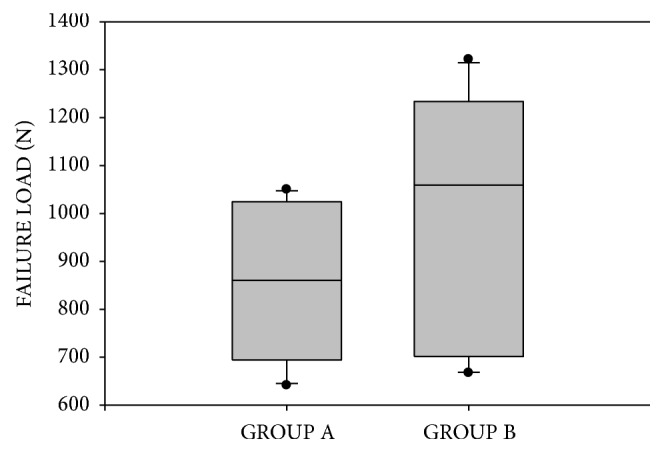
Boxplot for the results of fracture strength, including median, lower, and upper quartiles and minimum-maximum and outlier values.

The results of the failure load and the statistical analysis are summarized in Table 2. The median value of group A (860.5 N) was lower than group B (1059.2 N). However, statistical analysis showed no statistically significant difference between the two groups in the failure load (p = 0.273). In group B, half of the specimens exceeded the value of 1100 N, whereas in group A, four specimens presented values between 1000 and 1100 N. In both groups the lowest recorded values were above 600 N. The box plots of the results are presented in Figure 3.

**Figure 4:**
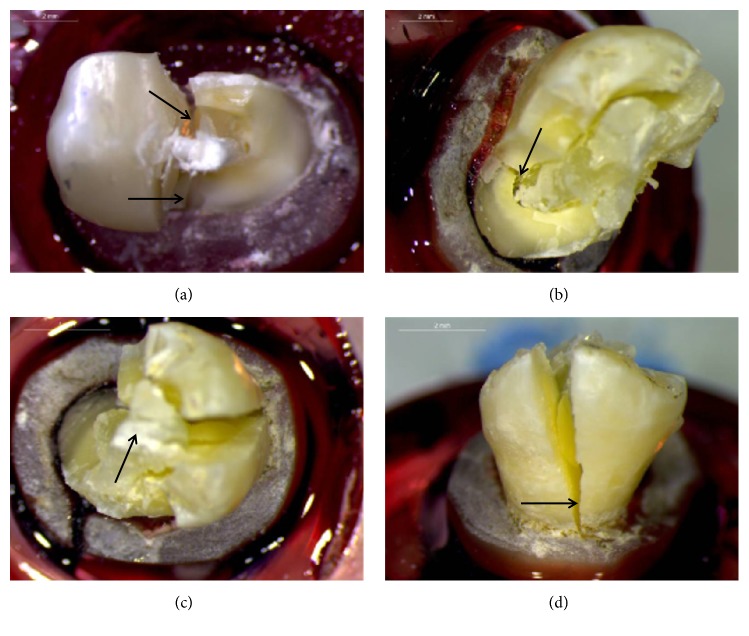
Fractured specimens of group A: (a) fracture of the post-retained restoration along with root fracture (arrows) and completely exposed post; (b) cleaved lingual wall (arrow) distal to the post; (c) fracture of buccal tooth wall, along with fracture and debonding of the restoration; (d) cleavage of the buccal wall through the middle level.

**Figure 5:**
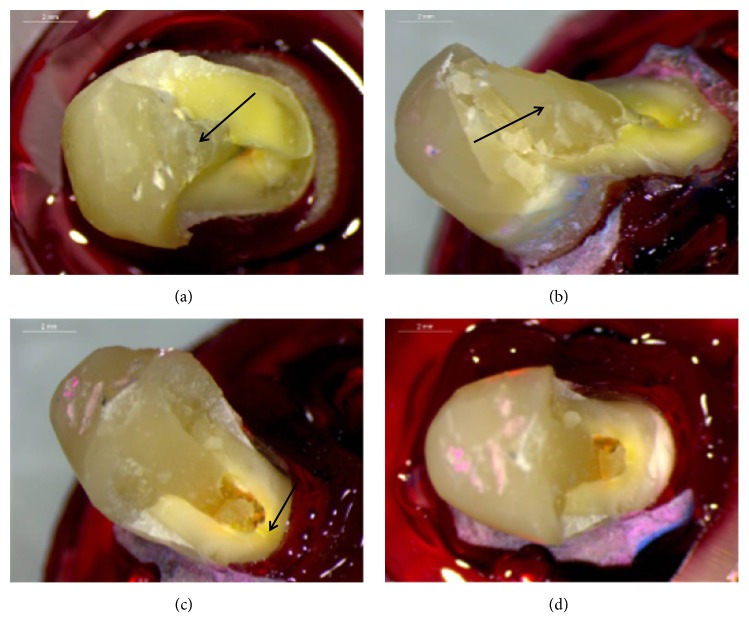
Fractured specimens of group B: (a) fracture of buccal, mesial, and distal walls (arrow indicates fiber-reinforced composite); (b) buccal tooth wall fracture with arrow showing the fiber-reinforced material; (c) lateral view of fracture of the buccal tooth wall, with minor secondary cracks (arrow) not extending to the margins; (d) top view of the same specimen (c) showing fracture of the buccal tooth wall extending to restoration margins.

Representative photographs of failed specimens are presented in Figures 4 and 5. The results of failure mode analysis are summarized in Table 3. In group A, 40% of the specimens revealed failure in the tooth crown walls and the restoration (type III failure), while the rest of the specimens (60%) showed catastrophic root fractures (type IV failure). Catastrophic root fractures were mostly combined with type III crown failures. On the contrary, in group B, no root fractures were identified in any of the specimens. Failures were equally distributed between type I and type III involving both crown tooth structure and the restorative material. In two cases of group B, debonding and cohesive fracture of the glass-FRC bulk fill liner was observed, while in all other cases failure was located within the glass-FRC bulk fill liner. The Chi-square test revealed statistically significant difference between groups A and B in the failure mode (p = 0.004).

## 4. Discussion

In the present study, the fracture resistance and mode of endodontically treated was evaluated in premolars restored either with glass-FRC posts and the PFC restorative or the glass-FRC bulk fill liner and the PFC restorative, after load cycling. The results of this in vitro study led to partial rejection of the null hypothesis. Although there was no significant difference in fracture strength between the groups tested, the specimens restored with the glass-FRC bulk fill liner and PFC restorative showed significantly less root fractures, considered as catastrophic failures, from restorations with the glass-FRC post and PFC restorative.

Maxillary two-rooted premolars of standardized size were selected for the study, since these teeth present an unfavorable anatomic shape, crown value, and crown/root proportion, making them more susceptible to fractures than other posterior teeth, when submitted to occlusal load application [32]. Also, a load cycling fatigue test was conducted, before final static loading up to fracture, in an attempt to mimic the actual function of mastication, even though laboratory simulations cannot accurately reflect the clinical conditions. A relatively low loading rate was used to provide time for elastic recovery and relaxation of such extended and complex restorations. Moreover, load cycling and loading up to fracture were performed along the longitudinal tooth axis, in order to concurrently load both tooth cusps. Preferential loading of the restoration, employing an inclined loading axis, was avoided to create an equivalent of simultaneous loading of the entire tooth crown. Finally, no intact teeth were used as controls, since comparison was limited only between treatments [33].

There are no similar studies available to compare the results of the present study. Hence, direct comparison of the results achieved from various laboratory studies evaluating the fracture resistance of ETT is not feasible, because of the differences in specimen type and size, tooth embedment methods, type and direction of load application, and aging conditions.

The ideal reconstruction of ETT should aim at improvement of their mechanical resistance and prevention of catastrophic failures. Traditionally, cuspal coverage along with cast post and core has been suggested as the only system to improve the ETT resistance and load distribution [34]. Recent developments in adhesive dentistry and minimally invasive concepts call for less destructive restorative techniques. Several studies have shown that direct PFC restorations may provide a significant improvement in the fracture resistance of posterior teeth when two or three walls are missing [35, 36]. Tooth structure preservation is directly correlated with fracture resistance and reduction of catastrophic failures [37, 38].

The glass-FRC post extension within the PFC restoration improves the ability of tooth-restoration complex to absorb occlusal loads and increase the resistance and retention of the ETT to masticatory forces [39, 40], probably as a result of more favorable distribution of functional stresses [41]. A positive effect of FRC posts in supporting PFC restorations has been reported in several laboratory and clinical studies [42–44]. Nevertheless, the results of other studies did not confirm such an effect, especially in premolars [45–47]. The loss of moderate dental structure and the presence of glass-FRC post restoration have been shown to reduce fracture resistance and create higher stress concentrations in the tooth-restoration complex. However, in cases with large loss of dental structure, glass-FRC posts reduced the incidence of catastrophic failures, although they did not reinforce the tooth-restoration complex [45]. More specifically, for endodontically treated premolars with residual wall thickness >2 mm, an intracuspal composite restoration supported by FRC post provided sufficient fracture resistance to occlusal loads, whereas in cases with residual wall thickness <2 mm, cuspal coverage through a composite resin restoration was mandatory, with or without a FRC post [36]. This implies that the most critical factor is the remaining tooth structure and not the FRC post reinforcement.

It has been postulated that any restoration without post space preparation and less sacrifice of residual sound tissue might result in greater resistance to fracture regardless of the degree of impairment of the dental structure [44]. Studies have shown that post space preparation not only weakens the tooth structure but also might lead to cracks and defects that can concentrate stresses and increase the possibility of root fracture and tooth loss [48].

In the present study, glass-FRC post placement (group A) did not protect the tooth from root fracture. Sixty percent of the teeth restored with a glass-FRC post and PFC fractured under the CEJ with vertical catastrophic root fractures, involving the pulp chamber floor at root bifurcation. All the rest showed mixed fractures of buccal tooth cusp and restoration, with post exposure to a various extent. No fracture or debonding of the adhesively bonded post to the root canal was identified in any of the specimens. This may imply that, despite the post bonding condition, a wedge-action cannot be avoided upon loading, with detrimental effects on root integrity. In group B, where the glass-FRC bulk fill liner was used under the PFC restoration, the median fracture strength was higher compared to that achieved by the glass-FRC post supported restorations, but this increase was not statistically significant. Evaluation of the fracture mode, though, showed no cases of catastrophic root fractures. This difference, which was statistically very significant, is probably the result of a much more favorable stress distribution provided by the specific restoration complex. The differences in the statistical ranking between fracture strength and mode may be explained by the contribution of the remaining tooth cusp in the overall strength. In 50% of group B specimens, only the buccal tooth cusp was fractured, indicating that the restoration was quite effective in distributing the fatigue stresses at the tooth crown. In group A specimens the high incidence of root bifurcation fractures was mostly combined with type III failures, revealing a stressful situation.

The glass-FRC used has been introduced a few years ago, as a bulk fill liner intended to be covered with a layer of a PFC. It is a combination of a semi-interpenetrating (IPN) matrix, short E-glass fibers randomly oriented, and inorganic particulate fillers. This FRC has been reported to exhibit improved physical and static/dynamic mechanical properties compared to classical PFCs, adequate degree of C=C conversion, and low polymerization shrinkage [49–53]. Short-fiber-reinforced composites have been evaluated in direct or indirect composite restorations of anterior and posterior vital and nonvital teeth [29, 33, 54]. It has been claimed that the function of short-fiber FRC liner is based on the support provided to the superficial PFC layer and an inhibition effect to crack propagation. The reinforcing effect of the fiber fillers is attributed not only to the favorable stress transfer characteristics from the polymer matrix to fibers, but also on the behavior of individual fibers as crack inhibitors. The stress transfer from polymer matrix to the fibers is a function of the fiber length, for optimal polymer reinforcement. The short fibers, incorporated in the glass-FRC bulk fill liner tested, are within the range of the critical fiber length (0.5-1.6 mm) to enable uniform stress distribution [55]. The high fibers volume fraction inside the restoration and layer thickness of the FRC liner further contribute to crack propagation inhibition and improved load-bearing capacity of the tooth-restoration complex [29, 33].

In the present study, the glass-FRC bulk fill liner was used as a 4 mm substrate under a 2 mm layer of the PFC restorative, with a 2 mm extension into the root canal. This design provides the advantages of a single-phase custom made fiber-reinforced short post, with full adaptation to the endo preparation geometry and a more predictable adhesive bonding to the cervical root canal dentin [56, 57]. Layering of the glass-FRC liner with a PFC is considered mandatory, because the presence of the short fibers fails to meet the criteria of wear resistance, roughness, and gloss set for PFC restoratives [58].

A superior fracture resistance and favorable fracture, coronal to the CEJ of endodontically treated posterior teeth restored with the glass-FRC bulk fill liner and a PFC, has been documented in several laboratory studies [29, 59, 60]. Moreover, further improvements in fractography were registered when the glass-FRC bulk fill liner was combined with a fiber-glass under CAD/CAM resin composite overlay restorations of endodontically treated molars, even though the load-bearing capacity was not improved significantly [33].

The present study focused on the fracture resistance and fracture mode of upper endodontically treated premolars with only one cusp missing, restored with direct PFC restorations supported either by glass-FRC post or glass-FRC bulk fill liner. According to the results, the combination of a glass-FRC bulk fill liner with a PFC restorative showed a promising performance regarding the fracture mode, providing a better reinforcing effect that could serve as a less invasive and time saving approach for the rehabilitation of posterior ETT, preventing thus catastrophic failures. More investigations need to be done to resolve specific issues such as the tooth type and size, cavity design, and remaining tooth structure, along with marginal leakage assessment and long-term performance of this type of restorations.

## 5. Conclusions

With the limitations of the present study, the following conclusions can be reached:

1. Median values of fracture load in N did not show any statistically significant difference between the two treatment modalities tested (group A: glass-FRC post; group B: glass-FRC bulk fill liner).
2. The failure mode of the fractured specimens presented statistically significant differences. In group A, 60% of the specimens demonstrated catastrophic root fractures (type IV failure mode) and 40% mixed fractures of residual tooth crown and restorative material (type III failure mode). In group B, no root fractures were found, with the failure modes equally distributed between type III (50%) and type I (50%), the latter including failure of residual tooth crown.
3. The glass-FRC bulk fill liner tested significantly modified failure mode, diminishing the catastrophic root fractures induced by FRC posts, at a similar or higher fracture load.

## Figures and Tables

**Table 1:** The products used for tooth restorations in groups A and B.

**PRODUCT /LOT**	**TYPE/COMPOSITION**	**MANUFACTURER**
G-CEM LinkAce A2/1309241	Self adhesive luting agent. *Resin:* DUDMA, GDMA, 10-MDP Catalysts: CHP, 2-tert-butyl-4,6-dimethylphenol. *Filler:* silanated glass (50–70 wt%)	GC Corporation, Tokyo, Japan

Glassix Radiopaque S1 13930	Glass-fiber post.	H. Nordin SA, Chailly Switzerland

Monobond-Plus R85603	Prehydrolyzed silane. 10-MDP, MPTMS, Disulfide dimethacrylate, Ethanol.	Ivoclar Vivadent, Schaan, Liechtenstein

GC Promotion Etchant -	40% phosphoric acid etching gel	GC Corporation, Tokyo, Japan

G-aenial bond Lot1308181	Self-etch adhesive. 4-MET, 10-MDP, Glycerol dimethacrylate, TEGDMA, water, acetone, initiators.	GC Corporation, Tokyo, Japan

EverX Posterior 1307124	Fiber-reinforced composite (FRC) *Resin: *semi-IPN: net-PMMA inter-net-poly(BisGMA): Bis-GMA, TEGDMA, PMMA *Fillers:* E-glass fiber, barium borosilicate (57% v).	GC Corporation, Tokyo, Japan

G-aenial Posterior A2/1306112	Particle-reinforced posterior resin composite (PFC). *Resin:* UDMA, dimethacrylate co-monomers, *Fillers:* Strontium and lanthanide containing prepolymerized fillers, silanated fluoroaluminosilicate glass, silica (65% v).	GC Corporation, Tokyo, Japan

**Table 2:** Results of failure load values in Newtons. Same superscript letters show median values with no statistically significant differences.

**GROUP**	**n**	**Median (N)**	**25**%** (N)**	**75**%** (N)**
A	10	860,5^a^	698	1024
B	10	1059,2^a^	708,5	1226,7

**Table 3:** The frequency of the failure modes identified. Type I: failure of remaining tooth crown walls only; Type II: failure of restoration only; Type III: mixed failure (I+II); Type IV: root fracture.

**Group**	**Type I**	**Type II**	**Type III**	**Type IV**
A	-	-	4 (40%)	6 (60%)^*∗*^
B	5 (50%)	-	5^*∗∗*^ (50%)	-

^*∗*^In all specimens this failure mode was combined with type III crown failures.

^*∗∗*^In two specimens fracture and debonding of the FRC composite occurred.

## Data Availability

All data supporting the results reported are available in technical report that has been composed and is available from the corresponding author upon request.
